# Ganglion Impar Neurolysis to Treat Refractory Chronic Pain From Vulvar Cancer: A Case Report

**DOI:** 10.7759/cureus.70128

**Published:** 2024-09-24

**Authors:** Oluwanifesimi Akinwamide, Asad Syed, Juhi Mehta, Marina Andrawis, Haijun Zhang

**Affiliations:** 1 Anesthesiology, Rutgers University New Jersey Medical School, Newark, USA

**Keywords:** ganglion impar, image-guided injection, neurolysis, refractory cancer pain, sacrococcygeal junction

## Abstract

Cancer pain treatment currently consists of the administration of pain medications, radiation therapy, tumor ablation as well as neurolytic plexus blocks. Neurolytic plexus blocks target both sensory afferent fibers and sympathetic fibers innervating visceral organs. Finding the right block for a specific type and location of cancer-related pain is crucial for the successful control of pain. One such example is a ganglion impar block for perineal pain. We report a case of a patient with chronic severe perineal pain from vulvar cancer and multiple surgeries, for whom a ganglion impar block followed by neurolysis provided significant pain relief.

## Introduction

According to the National Institutes of Health (NIH), approximately 20-50% of patients with cancer experience pain, and up to 33% of patients continue to experience cancer-related pain after curative treatments [1]. Modalities to treat cancer pain currently consist of the administration of pain medications enterally or intrathecally, external beam radiation therapy (EBRT), image-guided tumor ablation as well as neurolytic plexus blocks [2]. Neurolytic plexus blocks can target specific visceral sensory afferent fibers and sympathetic fibers to achieve adequate pain control [3]. The ganglion impar (also known as the sacrococcygeal ganglion) is a neural network containing both visceral sensory afferent fibers originating from the perineum, the distal section of the urethra, and the anogenital area as well as sympathetic fibers innervating the pelvic organs including the distal rectum and the distal urethra.

The blockade of the ganglion impar can directly interrupt the transmission of nociceptive signals from the innervated organs to the brain to relieve pain. It can also stop the activities of sympathetic nerves to limit the contribution of sympathetic components during pain signal transmission [3]. Over the years, using imaging guidance such as fluoroscopy or computed tomography (CT), many techniques have been developed to block the ganglion impar for pain relief [4]. Following the administration of local anesthetic, neurolysis of the ganglion is done via radiofrequency ablation (RFA) or chemicals such as alcohol or phenols. Ganglion impar neurolysis has been shown to effectively provide symptomatic pain relief to patients with perineal pain secondary to anogenital cancers and has significantly improved the quality of life of those patients [5-9].

With this case report, we aim to demonstrate the efficacy of ganglion impar neurolysis in controlling cancer-related perineal pain refractory to multifaceted management including surgeries and administration of pain medications.

## Case presentation

The patient was a 35-year-old female with a medical history of HIV, stage IB vulvar squamous cell carcinoma, and anal dysplasia status post (s/p) bilateral vulvectomy, radiation, and multiple ablations since 2012. She arrived at our pain clinic presenting with severe perineal pain, involving the vulva and anal area. She described the pain as a constant, burning pain with a visual analog score (VAS) score ranging from 7-10/10. On presentation, she had already taken a multimodal pain regimen including multiple opioid, non-opioid, and topical reagents for more than five years without adequate pain control. After discussing with the patient, given the failed modalities attempted thus far, we recommended a ganglion impar block with neurolysis as the next step. The patient agreed to proceed with the procedure after the risks and benefits of the procedure were explained to her. 

**Figure 1:**
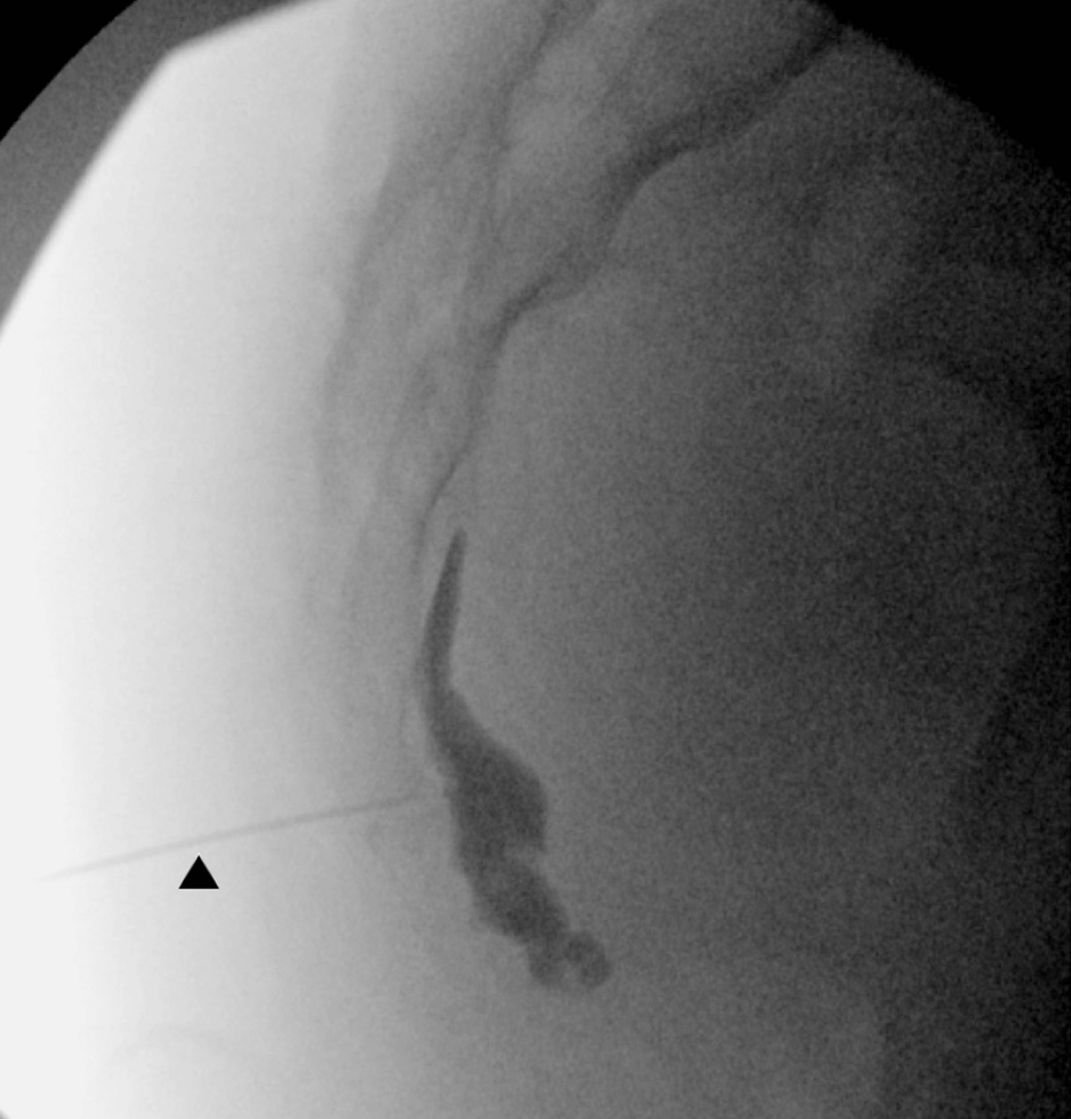
Fluoroscopic view of the ganglion impar block/neurolysis Lateral view of the sacrococcyx with the needle (arrowhead) at the sacrococcygeal segment showing contrast in the anterior edge of the sacrum where the ganglion impar is located.

Ganglion impar neurolysis was performed using a transsacrococcygeal approach under fluoroscopy guidance [9,10]. Briefly, a Quincke spinal needle was inserted into the sacrococcygeal junction under lateral fluoroscopic view and advanced until it entered and pierced through the sacrococcygeal disc segment and ligament to reach the anterior edge of the sacrum. As shown in Figure 1, the needle passed through the sacrococcygeal ligament and reached the anterior border of the vertebrae. The injection of the contrast showed the proper spread within the retroperitoneal space where the ganglion impar is located, confirming the correct placement of the needle. Next, 3 ml of 1% lidocaine was injected after negative aspiration. After confirming a significant pain relief from 6/10 to 1/10 following lidocaine administration, the mix of 4 ml of 0.25% bupivacaine and 4 ml of dehydrated alcohol was injected through the same needle. The needle was then removed after flushing with 0.5 ml 1% lidocaine. No complications were reported after the procedure. The patient reported her perineal pain decreased from 7/10 to 1/10, a roughly 85% reduction, for more than six months after the procedure. 

## Discussion

A ganglion impar block is a minimally invasive procedure with potential long-term advantages. Various techniques including trans-anococcygeal ligament and trans-sacrococcygeal approach have been described to achieve a successful block, but the trans-sacrococcygeal approach has been widely used [4]. The injection of local anesthetics blocks pain signals in the sacrococcygeal ganglion, a ganglion that contains autonomic nerve cells [3]. The blockade of the ganglion impar can be beneficial in alleviating visceral pain in the pelvic region, including the perineum, distal rectum, anus, distal urethra, vulva, and distal third of the vagina [4]. This block is especially useful in patients suspected to have pain with a sympathetic or neuropathic component. A ganglion impar block has clinical implications for patients with signs and symptoms, which include constant burning pain, cutaneous allodynia, and hyperalgesia. A ganglion impar block with neurolysis is typically performed under imaging guidance, such as fluoroscopy or computed tomography, and has a minimal rate of complications [4].

Theoretic risks include visceral damage (e.g., to the rectum), infection, bleeding, and diskitis, but no actual case of complications has been reported so far. Some patient factors such as severe coagulopathy, sepsis or local infection, and distorted anatomy due to trauma or surgery can limit the usage of ganglion impar blocks. The therapeutic effect of ganglion impar blocks/neurolysis can potentially last long [6,11,12], although there is no clinical data clearly showing which procedure can provide a longer effect. The block or neurolysis can also be repeated when necessary to treat the recurring pain. We recommend this treatment modality for patients suffering from chronic perineal pain secondary to malignancy. It can provide substantial symptomatic relief and improve quality of life, which is difficult to achieve by other treatments.

## Conclusions

Ganglion impar neurolysis can provide substantial pain relief of valvar and rectal pain in cancer patients. This procedure can be done safely under imaging guidance, and, thus, should be offered to patients early to improve their quality of life and potentially decrease the use of opioid medications. When necessary, this procedure can be repeated to treat the recurring pain. Although no actual case of complications has been reported so far, potential complications include visceral damage (e.g., to the rectum), infection, bleeding, and diskitis. In addition, patient factors that may increase the risk of complications or prevent the safe placement of needles can limit the usage of ganglion impar blocks/neurolysis.
